# Dynamic Notch signalling regulates neural stem cell state progression in the *Drosophila* optic lobe

**DOI:** 10.1186/s13064-018-0123-8

**Published:** 2018-11-22

**Authors:** Esteban G. Contreras, Boris Egger, Katrina S. Gold, Andrea H. Brand

**Affiliations:** 1 The Gurdon Institute and Department of Physiology, Development and Neuroscience, University of Cambridge, Tennis Court Road, Cambridge, CB2 1QN UK; 2 Present Address: Department of Biology, Zoology, University of Fribourg, Chemin du Musée 10, CH-1700 Fribourg, Switzerland

**Keywords:** Notch, Neuralized, Optic lobe, Neural stem cell

## Abstract

**Background:**

Neural stem cells generate all of the neurons and glial cells in the central nervous system, both during development and in the adult to maintain homeostasis. In the *Drosophila* optic lobe, neuroepithelial cells progress through two transient progenitor states, PI and PII, before transforming into neuroblasts. Here we analyse the role of Notch signalling in the transition from neuroepithelial cells to neuroblasts.

**Results:**

We observed dynamic regulation of Notch signalling: strong activity in PI progenitors, low signalling in PII progenitors, and increased activity after neuroblast transformation. Ectopic expression of the Notch ligand Delta induced the formation of ectopic PI progenitors. Interestingly, we show that the E3 ubiquitin ligase, Neuralized, regulates Delta levels and Notch signalling activity at the transition zone. We demonstrate that the proneural transcription factor, Lethal of scute, is essential to induce expression of Neuralized and promote the transition from the PI progenitor to the PII progenitor state.

**Conclusions:**

Our results show dynamic regulation of Notch signalling activity in the transition from neuroepithelial cells to neuroblasts. We propose a model in which Lethal of scute activates Notch signalling in a non-cell autonomous manner by regulating the expression of Neuralized, thereby promoting the progression between different neural stem cell states.

**Electronic supplementary material:**

The online version of this article (10.1186/s13064-018-0123-8) contains supplementary material, which is available to authorized users.

## Background

Throughout nervous system development, multipotent neural stem cells (NSCs) generate the vast diversity of neurons and glial cells present in the adult brain. In the mammalian brain, NSCs are a highly heterogeneous population that can alternate between active proliferative and quiescent states. Identifying the mechanisms that control NSC heterogeneity is essential for comprehending neurogenesis and brain regeneration.

**Fig. 1:**
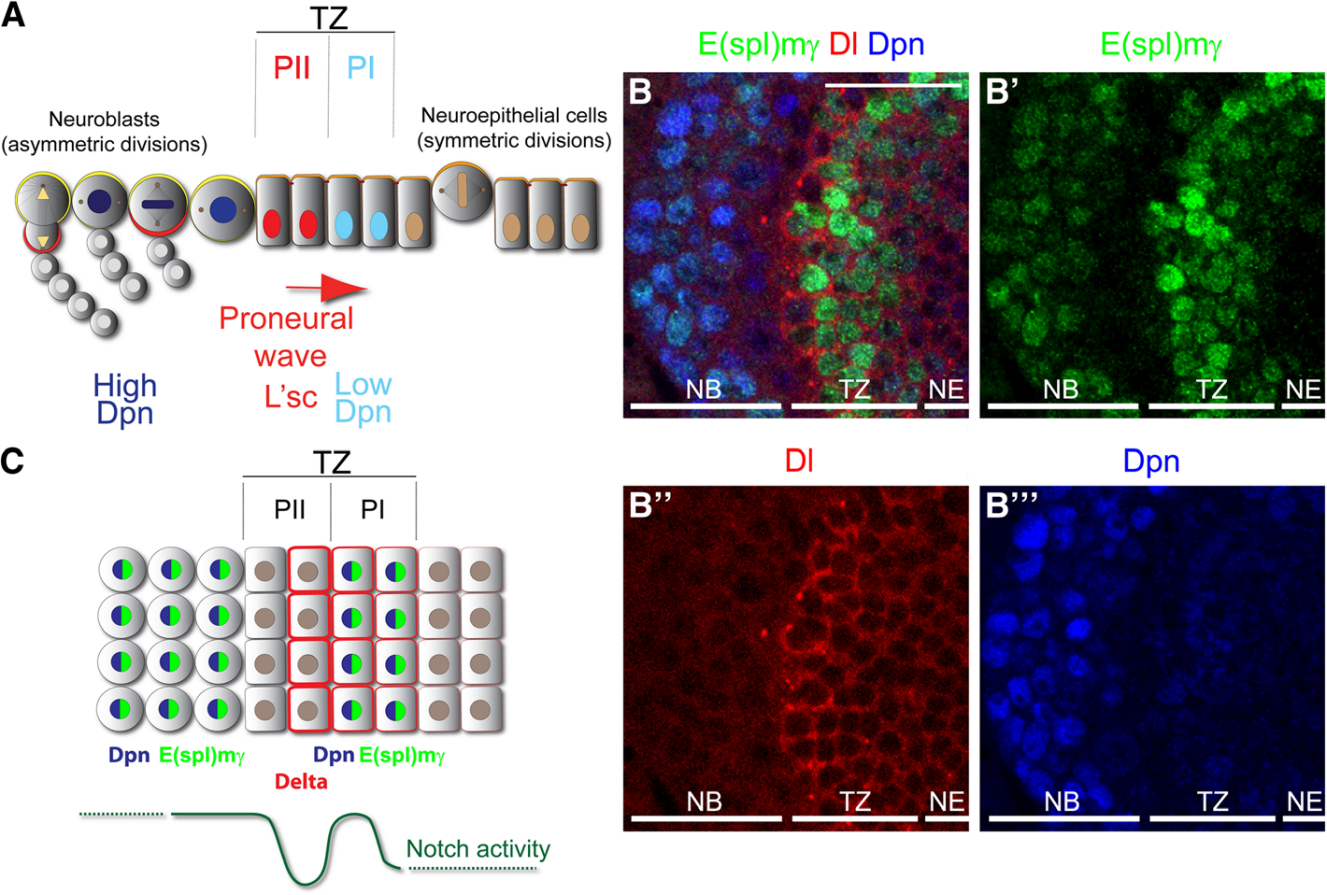
E(spl)mγ expression reports Notch signalling at the transition zone. (**a**) Schematic model of the optic lobe transition (TZ) between NE cells into NBs. NE cells divide symmetrically to amplify their pool and transform into PI progenitors, expressing low levels of nuclear Dpn (blue). PI progenitors transform into PII progenitors, characterised by the expression of L’sc (red), and PII progenitors transform into NBs that divides asymmetrically and generate differentiated progeny. Modified from [8]. (**b-b”’**) Immunostaining of the optic lobe transition zone expressing the Notch reporter (**b’**) E(spl)mγ-GFP (green) and stained for (**b”**) Dl (red) and (**b”’**) Dpn (blue). (**c**) Schematic model of Notch signalling activation at the optic lobe transition zone, showing two peaks of Notch signalling activation in PI progenitors and in NBs. Scale bars are 20 μm

The *Drosophila* optic lobe, which shares many of the features of neurogenesis in the mammalian cerebral cortex [1], is a simple model for understanding NSC diversity. *Drosophila* and vertebrate neuroepithelial (NE) cells exhibit states of amplification and differentiation [2–4], as well as interkinetic nuclear migration [5]. The optic lobe develops from neuroepithelial cells that divide symmetrically, increasing their number, and then transform into neuroblasts (NBs) at a region called the ‘*transition zone*’ (Fig. 1B). Asymmetrically dividing neuroblasts self-renew and generate ganglion mother cells (GMCs) that divide once more to generate postmitotic neurons and/or glial cells [3, 4, 6]. The optic lobe transition zone is characterised by the progressive change of NSC states from neuroepithelial cells into neuroblasts, via two intermediate types of neuronal progenitors: PI and PII. PI progenitors express low levels of the neuroblast marker, Deadpan (Dpn), while PII progenitors are defined by the expression of the proneural gene, Lethal of scute (L’sc) [6–8]. In order to generate the optic lobe retinotopic map, a strict regulation of neuroepithelial cell amplification and state progression is necessary. The transition zone requires the action of several signalling pathways to regulate the expression of L’sc in a dynamic pattern described as a proneural wave [6–9] (Fig. 1a). These signalling pathways control NSC state progression, however, how they are precisely integrated is not well understood.

The Notch signalling pathway is a key regulator of cell-cell communication required for stem cell self-renewal and differentiation [10]. When either Delta or Serrate binds to Notch on a neighbouring cell, the Notch intracellular domain (NICD) is cleaved and translocated to the nucleus, promoting the expression of target genes [11]. Several studies indicate that Notch signalling is key for NSC maintenance in the developing and adult brain [10, 12, 13], however, Notch signalling can promote both NSC proliferation and quiescence depending on the signalling context [14]. In the *Drosophila* optic lobe, Notch signalling regulates neuroepithelial cell amplification and fate maintenance in a manner similar to vertebrate NSCs. Notch signalling is activated across the entire neuroepithelium and loss of Notch function induces premature transformation of neuroepithelial cells into neuroblasts [7, 15–21]. Furthermore, ectopic activation of Notch signalling is sufficient to delay the transformation of neuroepithelial cells into neuroblasts [7, 19]. Although Notch function is required to maintain neuroepithelial cell fate, its signalling is essential for neuroblast proliferation [22, 23]. How this dual role of Notch signalling is regulated to allow the progressive change from neuroepithelial cells into neuroblasts is not completely understood.

Here we show that the ligand Delta (Dl) and the E3 ubiquitin ligase Neuralized (Neur) have key roles in the neuroepithelial cell to neuroblast transition. Dl and Neur are required for Notch signalling at the transition zone. We find that L’sc is sufficient to induce *neur* expression and the formation of ectopic transition zones. We propose a backward relay model in which L’sc controls cell autonomous as well as cell non-autonomous mechanisms to drive the neuroepithelial to neuroblast transition.

## Methods

### Drosophila lines

The following fly genotypes were used: *E(spl)mγ-GFP* [24], *neur-lacZ/TM6B* [25], *UAS-Dl* [26], *UAS- N*^*FL*^ [27], *UAS-N*^*ICD*^ [28], *hs-Flp; UAS-L’sc* [29]. Flip-out clones were used for misexpression and they were generated using *yw, hs-Flp; tub > Stop > GAL4*, *UAS-nls-lacZ/Cyo*, *Dfd-EYFP* or *Act5c > Stop > GAL4*, *UAS-GFP*; *neur-lacZ/TM6B*. Mutant clones were generated using *hsFlp;; FRT82B, Ubi-RFP/TM6B* and *FRT82B, Dl*^*rev10*^*/TM6B* [30] or *FRT82B, neur*^*1*^*/TM6B* [31].

### Generation of mutant and misexpression clones

Flip-out clones and mutant clones were induced 24 h after larva hatching (ALH) and brains were dissected and stained 78 h ALH. Flip-out clones were induced for 10 min at 37 °C, whereas for mutant clone generation larvae were heat-shocked for 30 min at 37 °C. Larvae were kept at 25 °C.

### Immunofluorescence

Larval brains were fixed and stained as previously described [32]. The following primary antibodies were used: rabbit anti-Ase (1:1000 from Y.N. Jan), chicken anti-β-gal (1:100 abcam), mouse anti-Dl (1:100, C594.9B Developmental Studies Hybridoma Bank, DSHB), guinea pig anti-Dpn (1:5000, from J. Skeath), chicken and rabbit anti-GFP (1:2000 abcam), rat anti-L’sc (1:5000) and anti-Notch (1:50, C17.9C6 DSHB). Alexa Fluor conjugated secondary antibodies were diluted 1:200 (Molecular Probes, Invitrogen). Primary and secondary antibodies were incubated at 4 °C overnight.

### In situ hybridisation

Probes were generated by PCR amplification from a embryonic cDNA library. Reverse primers contained the T7 polymerase promoter. *Neur* probe were generated using the following primers: Fw 5′- ACTCGCAATCAAACCTACTAAAGC-3′ and Rv 5′- CAGTAATACGACTCACTATTA AAGTGTAATTTAAAATGCGGCTTC-3′. For *tom* probe we used: Fw 5′- AAATCTCAACAATCCTCAACACAA-3′ and Rv 5′- CAGTAATACGACTCACTATTA TACGAAGACCCTAACAAACAAACA-3′ [16].

in situ hybridisation was performed using a standard protocol. Briefly, third instar larval brains were fixed in 4% Formaldehyde in 1X PBS, washed with PBT (1X PBS, 0.1% Tween-20) and permeabilised using 50 μg/mL Proteinase K. Probes were hybridised at 55 °C, brains were blocked 30 min using 10% normal goat serum and incubated with anti-digoxigenin AP (1:2,000 Roche) for 2 h. Staining was performed using NBT/BCIP.

### Imaging

Images were acquired using a Leica SP5 confocal microscope or a Zeiss Axioplasm microscope with a Leica DFC420C camera. Images, diagrams and figures were assembled using Fiji, adobe Photoshop CS2 and Illustrator CS3.

## Results

### E(spl)mγ reports Notch signalling in the optic lobe transition zone

Notch signalling is necessary to maintain both neuroepithelial cell and neuroblast fates. To understand the regulation of Notch signalling during the transition of neuroepithelial cells to neuroblasts, we searched for a Notch reporter that precisely reflects the activation of the pathway. Several Notch reporters have been characterised as expressed in neuroepithelial cells and neuroblasts, however, most of these express GFP or lacZ under the control of a Notch target gene promoter. Due to the stability of GFP and β-galactosidase, these reporters do not reflect rapid changes in Notch signalling. To overcome this, we used the E(spl)mγ-GFP reporter (hereinafter referred as E(spl)mγ) that contains the E(spl)mγ promoter and coding sequence fused to GFP, reflecting the dynamics of E(spl)mγ protein half-life and turnover [24].

E(spl)mγ was expressed at high levels at the transition zone (Fig. 1b-b”’). Interestingly, E(spl)mγ expression was completely downregulated before neuroblast formation and then reexpressed in neuroblasts (high Dpn-positive cells, see Fig. 1b’,b”’). Notch signalling downregulation correlated with high levels of Dl (Fig. 1b,b”). This expression pattern suggests that Notch signalling is highly active in PI progenitors, blocked after PII induction and restored upon neuroblast transformation (Fig. 1b).

**Fig. 2:**
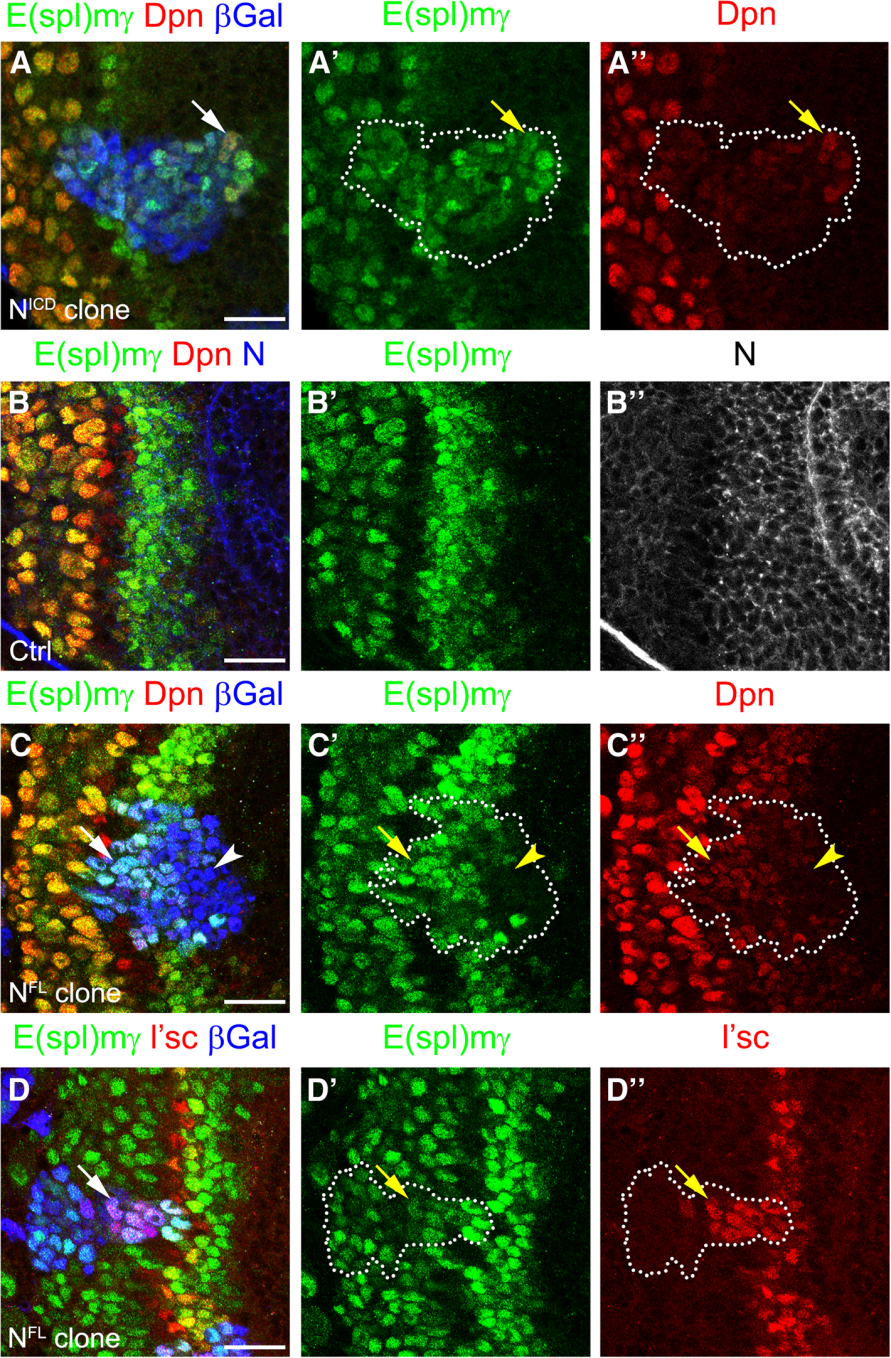
Notch signalling regulates PI progenitor fate and prevents PII progenitor conversion into neuroblasts. (**a-Aa”**) Staining of clone misexpressing the N^ICD^ in the optic lobe transition zone. Clone was marked by β-gal expression (blue) and marked by dotted lines; E(spl)mγ expression in green, and Dpn in red. (**b-b”**) Wild-type brain transition zone stained for E(spl)mγ in green, Dpn in red and Notch receptor in blue (**b**) or grey (**b”**). Arrows indicate the end of Notch receptor and Notch signalling activation (**c-d”**) Staining of clones misexpressing a full length Notch receptor (N^FL^) for (**c - d”**) E(spl)mγ in green, Dpn in red (**c, c”**) and L’sc in red (**d, d”**). Arrows indicate E(spl)mγ activation after PI progenitor formation and (**d-d”**) a delay in PII progenitor transformation into NBs. Arrowheads show cells in the clone that do not activate Notch signalling (**c-c”**). Scale bars are 20 μm

In order to confirm that E(spl)mγ expression was regulated by Notch signalling, we generated clones misexpressing the intracellular domain of Notch (N^ICD^), which activates Notch signalling in a cell autonomous manner. N^ICD^ clones marked with β-gal expressed high levels of E(spl)mγ, confirming that the reporter was activated by Notch signalling. Furthermore, N^ICD^ clones also expressed low levels of Dpn suggesting that PI progenitor fate is induced by Notch signalling (see arrow in Fig. 2a-a”). Therefore, E(spl)mγ expression reflects the dynamic activity of Notch signalling at the transition zone.

### Notch levels control signalling activity at the transition zone

The expression of E(spl)mγ suggested a precise regulation of Notch signalling. Notch signalling was quickly blocked in one or two cells before neuroblast transformation and activated again in neuroblasts. Given that E(spl)mγ-negative cells were in direct contact with Dl-positive cells (Fig. 1b), we hypothesised that Notch signalling was regulated by the levels of receptor. We analysed the expression of Notch receptor at the transition zone (Fig. 2b,b”). Although Notch was expressed in all neuroepithelial cells, the E(spl)mγ reporter was activated only at the transition zone (Fig. 2b,b”). Interestingly, Notch and E(spl)mγ levels were reduced together at the end of the transition zone (see arrow Fig. 2b-b”) and increased after neuroblast transformation, suggesting that Notch signalling is regulated by the levels of expression of Notch.

To assess whether downregulation of Notch is the main mechanisms for blocking Notch signalling at the transition zone, we generated clones expressing a full length form of Notch (N^FL^). N^FL^ clones activated the E(spl)mγ reporter only at the transition zone, while no E(spl)mγ expression was observed in clones in the middle of the neuroepithelium, where Dl is not expressed (Fig. 2c-c”). However, N^FL^ clones that crossed the transition zone maintained expression of E(spl)mγ and low levels of Dpn, suggesting that Notch signalling was active and induced PI progenitor fate (see arrow in Fig. 2c-c”). Additionally, N^FL^ clones that crossed the transition zone maintained L’sc expression, delaying the transformation into neuroblasts (see arrow in Fig. 2d-d”). These results suggest that Notch expression is rapidly downregulated in order to block its signalling, which is necessary to allow the precise transition from PII progenitors into neuroblasts.

### Delta activates Notch signalling inducing the formation of PI progenitor state

**Fig. 3:**
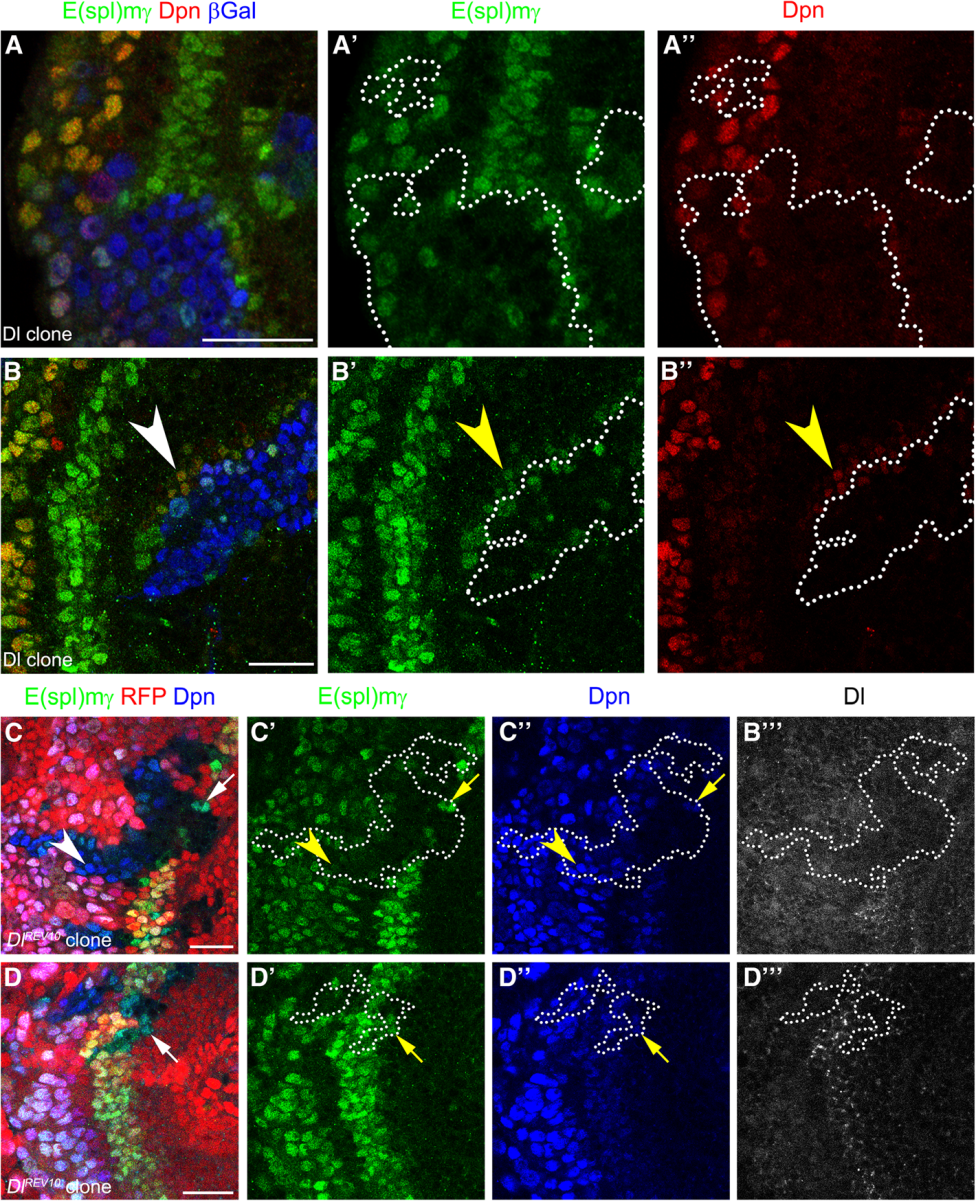
Delta necessary ans sufficient for Notch signalling inducing PI progenitor formation. (**a-b”**) Immunostaining of Dl misexpressing clones, E(spl)mγ in green, and Dpn in red. Clones were marked by β-gal staining in blue and dotted line. Arrowheads show E(spl)mγ activation in clone neighbouring cells. (**c-d”’**) *Dl*^*rev10*^ mutant clones stained for E(spl)mγ in green, Dpn in blue, and Dl in gray. Clones were marked by the absence of RFP expression and dotted lines. Arrows show E(spl)mγ expression inside mutant cells that were in contact with wild-type cells. Arrowheads show NBs not expressing E(spl)mγ. Scale bars are 20 μm

To understand the role of Dl at the transition zone, we generated Dl misexpression clones and assessed E(spl)mγ expression. Dl misexpression blocked E(spl)mγ expression at the transition zone (Fig. 3a-a”), but activated E(spl)mγ expression and induced low levels of Dpn in neighbouring wild-type cells (see arrowheads in Fig. 3b-b”). This result suggests that Dl can activate Notch signalling, inducing PI progenitor fate in a non-cell autonomous manner, but that high levels of Dl block Notch signalling in a cell autonomous manner. However, we did not observe high levels of E(spl)mγ and Dpn surrounding the clones, suggesting that ectopic PI progenitors generated by Dl misexpression might not be competent to transform into neuroblasts.

To characterise further the role of Dl in Notch signalling, we analysed *Dl* mutant clones. Mutant clones for a null allele of *Dl* (*Dl*^*rev10*^) [30] were generated by mitotic recombination and marked by the absence of RFP expression. These clones had no detectable Dl (Fig. 3c-c") and the levels of the E(spl)mγ reporter and Dpn were decreased (see arrow in Fig. 3c-c”), suggesting that Dl is necessary for Notch signalling and PI progenitor induction at the transition zone. Interestingly, E(spl)mγ expression was also downregulated in mutant neuroblasts (see arrowhead in Fig. 3c-c’). Small *Dl* mutant clones were not affected and showed normal E(spl)mγ expression, suggesting that wild-type cells can rescue Notch signalling in a non-cell autonomous manner (Fig. 3d-d”’). Non-cell autonomous activation could also be observed in mutant cells of larger clones, which were adjacent to Dl expressing wild-type cells (see arrows in Fig. 3d-d”’). Together these results strongly suggest that Dl is the major ligand for Notch activation and PI progenitor state induction at the transition from neuroepithelial cells to neuroblasts.

### Neuralized is required for Notch signalling at the transition zone

**Fig. 4:**
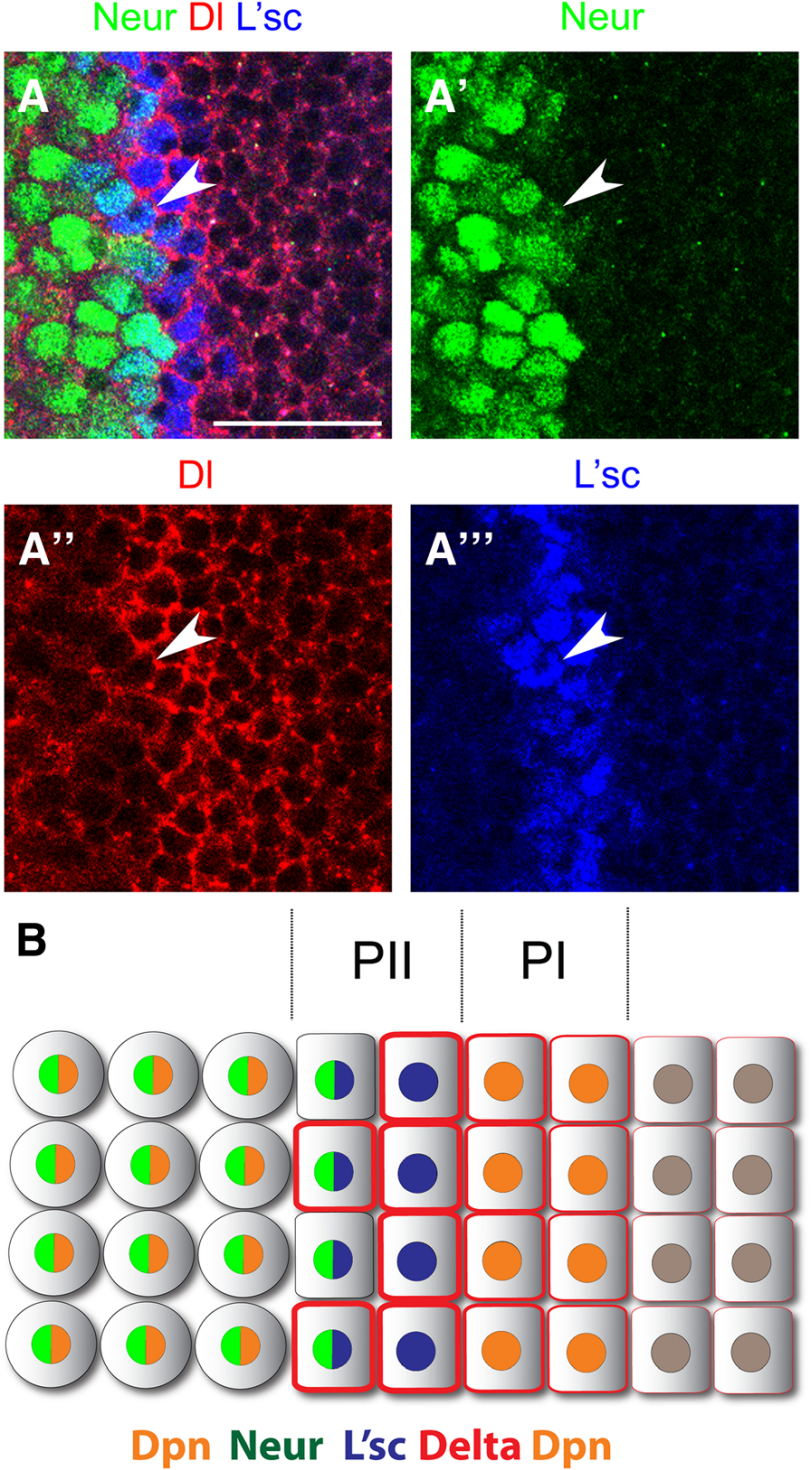
*neuralized* is expressed in PII progenitors and in optic lobe neuroblasts. (**a**) Immunostaining of *neur-lacZ* larval brains for β-gal/*neur* in green, Dl in red and L’sc in blue. Arrowheads show PII progenitor expressing *neur*, Dl and L’sc. (**b**) Schematic representation of *neur* expression during the transition between NE cells into NBs. Scale bars are 20 μm

The E3 ubiquitin ligase *neuralized* (*neur*) [33–35] promotes endocytosis of the Dl ligand, activating Notch signalling in neighbouring cells [36, 37]. As Neur function has not been assessed during optic lobe development, we decided to investigate whether it participates in the regulation of Notch signalling at the transition zone. We used a *lacZ* insertion in the *neur* locus (*neur-lacZ*) as an expression reporter during the transition from neuroepithelial cells into neuroblasts. [25]. *neur-lacZ* expression was observed at the end of the transition zone and in optic lobe neuroblasts. Neur is initiated in the second of the L’sc expressing PII progenitors, just prior to their transformation into Dpn positive neuroblasts (Fig. 4a). These medial PII progenitors also expressed Dl (see arrowhead in Fig. 4a-a”’), but at lower levels than the most lateral PII progenitor. We observed high levels of *neur* mRNA at the transition zone, in a pattern complementary to *twin of m4* (*tom*) expression, a Notch target gene expressed across the neuroepithelium [16] (Additional file 1: Figure S1). Therefore, *neur* is expressed in medial PII progenitors and in optic lobe neuroblasts (Fig. 4b).

**Fig. 5:**
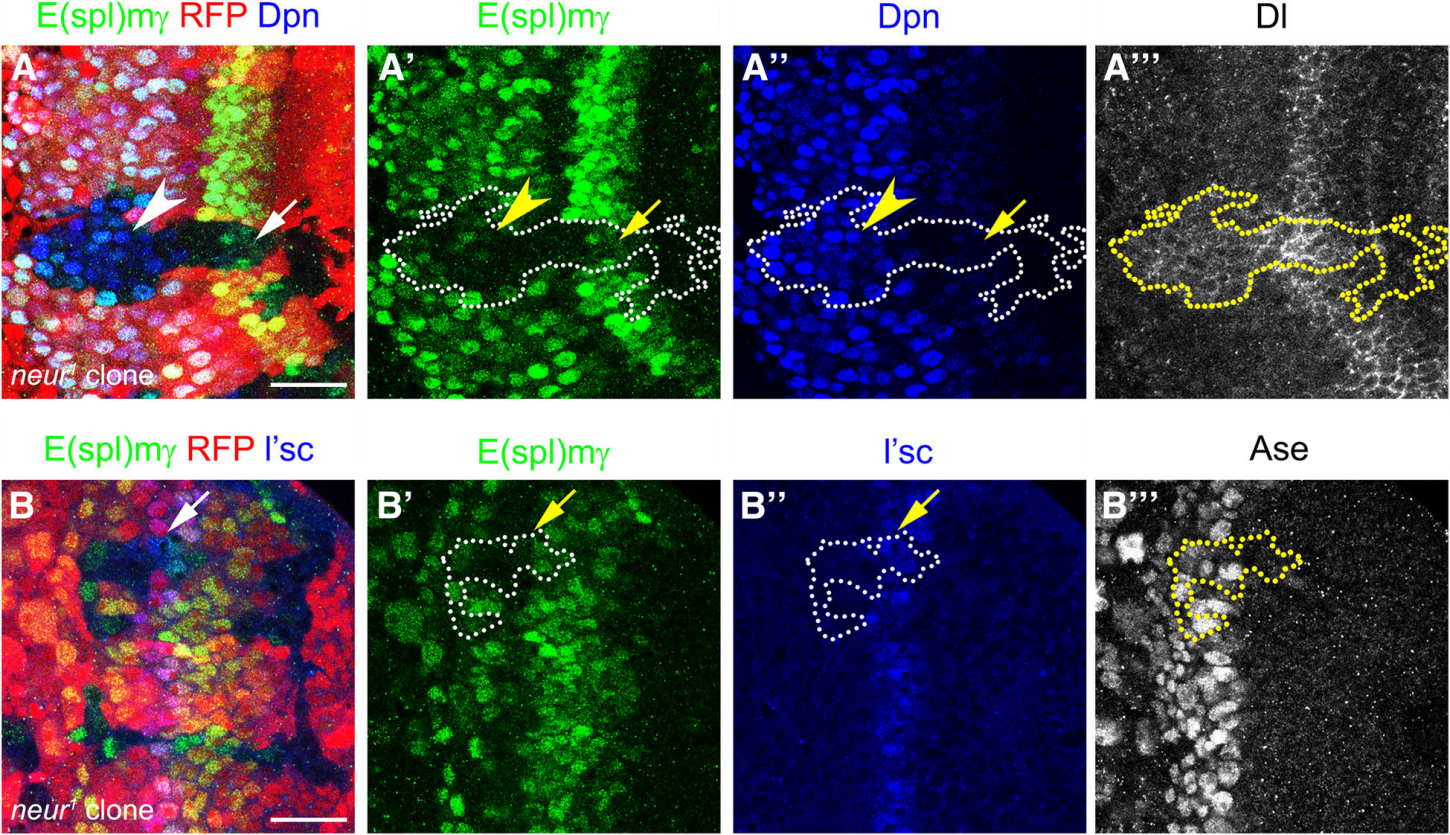
Notch signalling activation requires Neuralized function at the transition zone. (**a-b”’**) *neur*^*1*^ mutant clones stained for E(spl)mγ in green, (**a,a”**) Dpn in blue, (**b,b”**) L’sc in blue, (**a”’**) Dl in gray and (**b”’**) Asense (Ase), as a neuroblast marker, in gray. Clones were marked by the absence of RFP expression and dotted lines. (**a-a”**) Arrows show decrease in E(spl)mγ staining in PI progenitors and arrowheads in NBs. (**b-b”**) Arrows pointed L’sc-positive PII progenitor inside *neur* mutant clone. Scale bars are 20 μm

To assess Neur function, we generated *neur* mutant clones using a null allele (*neur*^*1*^) [31]. Mutant clones showed a reduction in E(spl)mγ expression in a cell autonomous manner (Fig. 5a-a”’) resembling *Dl* mutant clones (compare to Fig. 3a-a”’). The reduction in E(spl)mγ expression was observed in optic lobe neuroblasts (69.7% of clones, 23/33, see arrowhead in Fig. 5a-a”) and also in PI progenitors (52.0% of clones, 13/25), in which Dpn levels were also reduced (see arrow in Fig. 5a”). In addition, while L’sc levels were normal in *neur* mutant cells, Dl levels were upregulated in clones at the transition zone and in Dpn-positive neuroblasts (Fig. 5a”’ and 5b”). This suggests that *neur* is required to activate Dl-mediated Notch signalling and to induce PI progenitor state in the neuroepithelial to neuroblasts transition zone.

### Lethal of scute is sufficient to induce *neuralized* expression and to generate ectopic transition zones

**Fig. 6:**
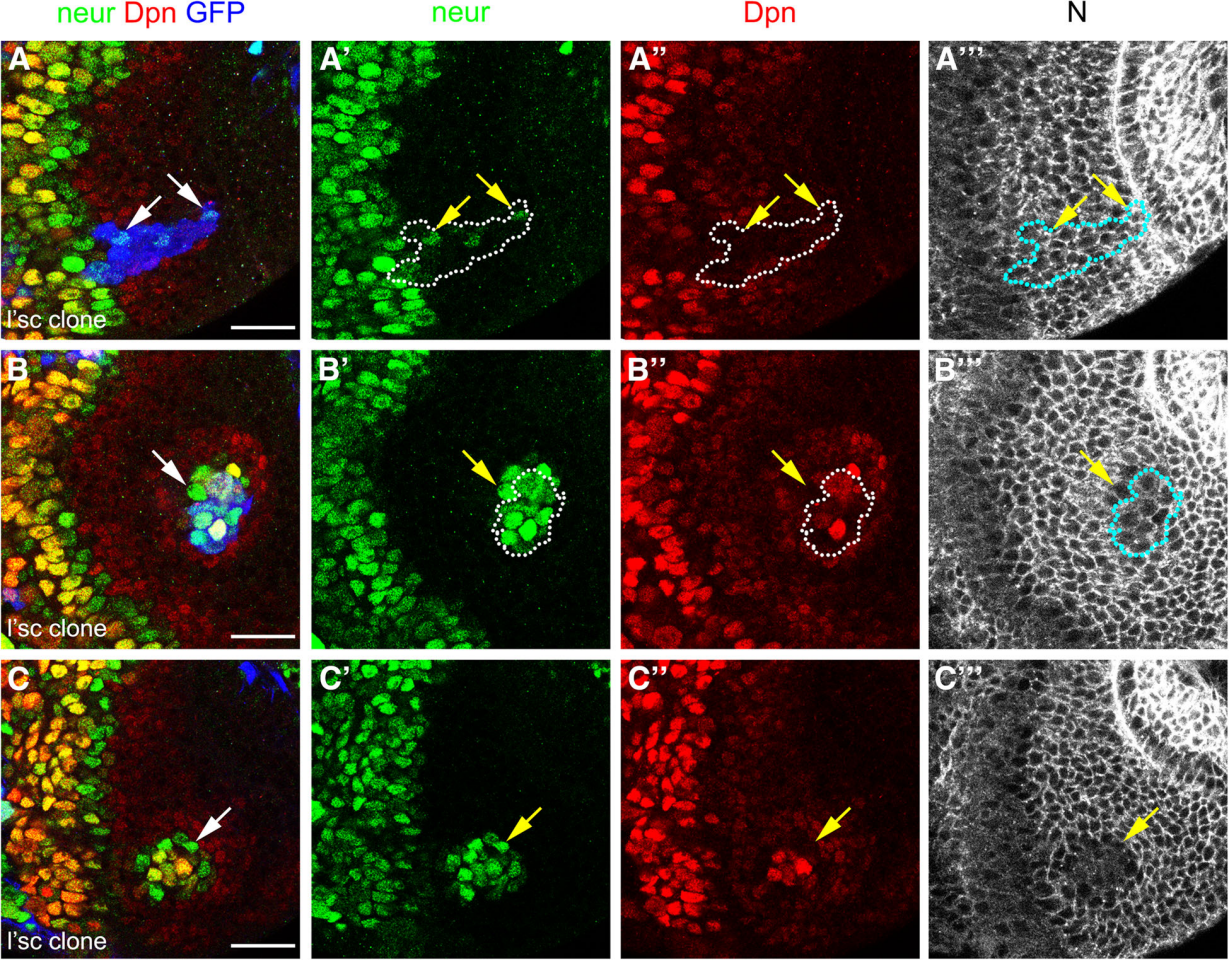
Lethal of scute regulates *neutralized* expression and generates ectopic transition zone in a cell non-autonomous manner. (**a-c”’**) Immunostaining of L’sc misexpressing clones in *neur-lacZ* larval brain for β-gal/*neur* in green, Dpn in and Notch in gray. Clones were marked by GFP expression in blue and dotted lines. Arrows show ectopic activation of *neur* expression (**a-a”’**) inside and (**b-c”’**) outside L’sc misexpressing clones. Note that in (**c-c”’**) there is no NE cell misexpressing L’sc (no GFP expression, blue). Scale bars are 20 μm

*neur* was expressed preferentially in the L’sc-positive PII progenitors closest to neuroblasts (Fig. 5). PII progenitor fate is defined by the expression of L’sc [7], hence we hypothesised that L’sc regulates *neur* expression in order to activate Notch signalling and induce PI progenitor fate. To test this, we generated L’sc misexpression clones outside the transition zone. L’sc misexpression was sufficient to induce *neur* expression in neuroepithelial cells (see arrows in Fig. 6a-a”’). Remarkably, L‘sc misexpression generated ectopic transition zones in the neuroepithelium (Fig. 6b). These clones showed high levels of Dpn and Neur, and a decrease in Notch receptor levels, demonstrating that ectopic neuroblasts were generated by L’sc misexpression (Fig. 6b-b”’).

Interestingly, *neur* expression was also observed outside the clones (see arrow in Fig. 6b-b”) as were PI progenitors (low Dpn-positive cells; Fig. 6b”). In some cases, L’sc misexpressing cells were found deep inside the optic lobe (see blue clone in Additional file 2: Figure S2), suggesting that the L’sc misexpressing cells initiated the ectopic transition zone and then delaminated from the neuroepithelium after neuroblast transformation. The ectopic transition zones remained in the neuroepithelium after the clones had delaminated (note lack of GFP expression in Fig. 6c). These ectopic transition zones contained Dpn-positive PI progenitors, Neur-positive PII progenitors and Dpn-positive/Neur-positive neuroblasts.

We conclude that the induction of L’sc within the neuroepithelium is sufficient to induce *neur* expression and to generate ectopic transition zones containing PI and PII progenitor states in a non-cell autonomous manner. Remarkably these ectopic transition zones are maintained and continue to generate neuroblasts.

## Discussion

Notch signalling acitivity is dynamically regulated in the transition zone. The E(spl)mγ reporter is highly expressed in PI progenitor cells, downregulated in PII progenitor cells and upregulated again in neuroblasts [8, 38]. Here, we demonstrate that the ligand Delta and the E3 Ubiquitin ligase Neur are required in PII progenitor cells to activate Notch signalling in neighbouring PI progenitors. We also show that Neur expression is induced by the proneural factor L’sc, which is able to induce the entire transition zone.

### A switch from serrate to Delta mediates Notch signalling in the progression of neural stem cell states

Notch mutant clones are extruded from the neuroepithelium and prematurely transform into neuroblasts at ectopic positions [16]. Interestingly, *Dl* mutant clones in the lateral neuroepithelium do not phenocopy these *Notch* null mutant clones [7]. This suggests that Dl is not required for Notch signalling in more lateral proliferating neuroepithelial cells and that Notch is activated by a different ligand. Indeed, Perez-Gomez et al. [15] showed that glial cells adjacent to the neuroepithelium activate Notch signalling via the ligand Serrate (Ser). Ser is necessary for neuroepithelial cell proliferation and for preventing PII progenitor formation [15]. Hence, we favour a model in which Notch signalling induce by Serrate maintains neuroepithelial cells in a proliferating state, while Notch signalling induced by Delta initiates PI progenitor formation and the neuroepithelial cell to neuroblast transition.

The differential expression of Notch signalling modulators, such as the protein Canoe (Cno), may explain preferential binding for one of the two ligands. Canoe stabilises the Notch receptor at adherens junctions and promotes binding to Ser from glial cells [15]. The E3 ubiquitin ligase, Mind bomb, is required for the activation of Ser while Neur controls the activity of Delta [39].

**Fig. 7:**
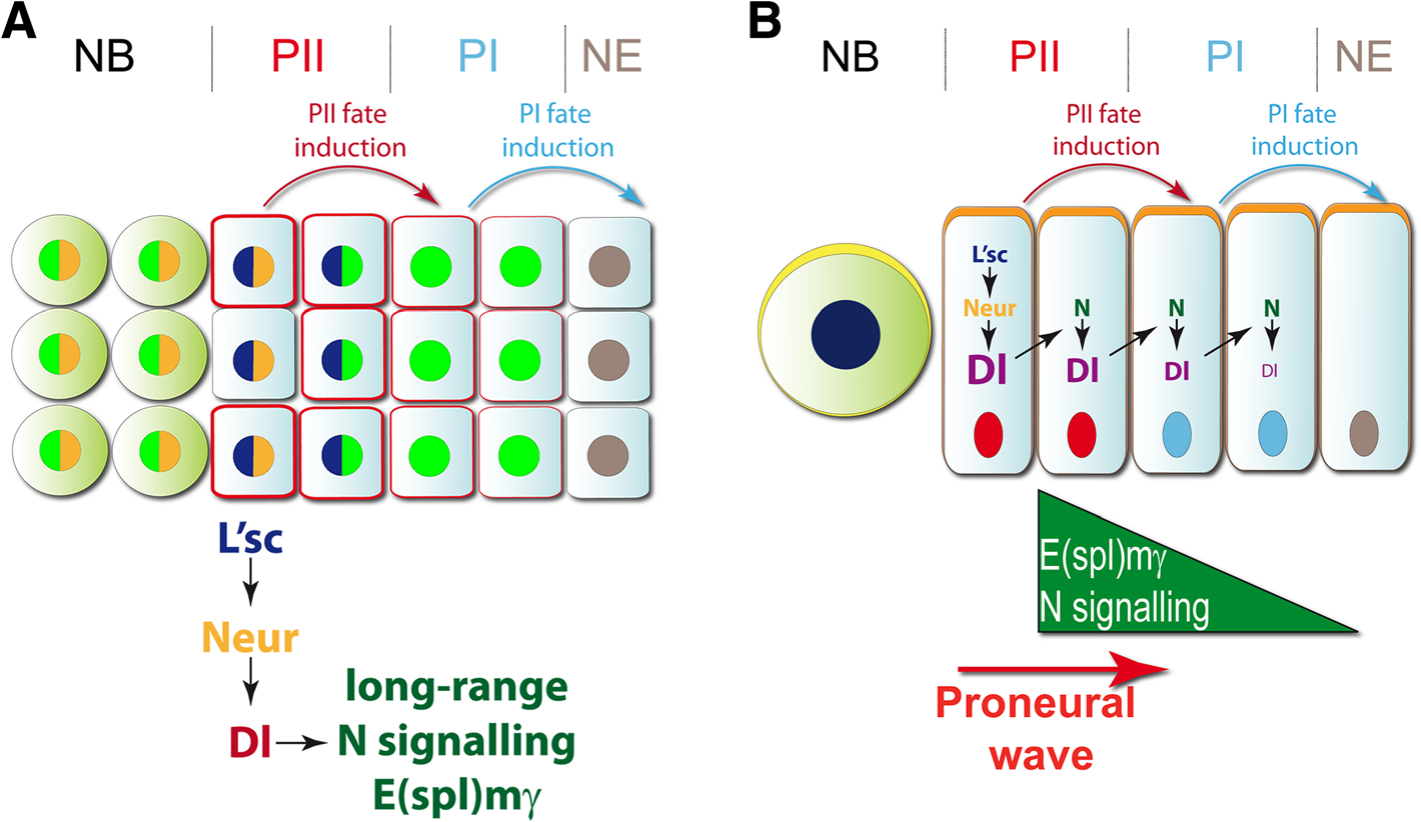
Working models of Notch signalling during the transition of neural stem cell states. Two models showing the progression of the transition between NE cells into NBs. **a** Long-range activation of Notch signalling in PI progenitors can be controlled by L’sc in PII progenitors. L’sc regulates *neur* expression that activates Dl function. **b** Activation of Notch signalling is regulated by L’sc-positive/Neur-positive/Dl-positive PII progenitors inducing Dl expression in the closer neighbour and generating a gradient of E(spl)mγ expression in PI progenitors. In both models, PII progenitors are able to induce the PII fate in PI progenitor, while PI progenitors promote NE cells transformation intro PI state. When PII progenitors convert into NB, PI progenitors replace PII progenitors and NE cells convert into PI progenitors, promoting the progression of the proneural wave

We show that *neur* expression is restricted to PII progenitors cells closest to the neuroblasts (Fig. 4). However, the loss of *neur* affects cells that are not immediate neighbours, the PI progenitors, implying that Delta-Notch signalling may work over a distance. Membrane protrusions may allow Dl to activate N signalling at a distance, as has been described during bristle development [40, 41] (Fig. 7a). Alternatively, it has been shown that Notch signalling promotes Dl expression [20]. This positive feedback loop may allow the initial Neur activity to propagate in a non-cell autonomous manner, generating a gradient of Notch signalling (Fig. 7b).

### A backward relay mechanism controls changes in neural stem cell states

Neuroepithelial cells progress through two transient progenitor states prior to transforming into medulla neuroblasts [6–8]. Here we show that PII progenitors can be further subdivided into L’sc, high Delta and L’sc, Neur expressing cells (Fig. 7). Two opposing signalling pathways control the medial to lateral progression of the proneural wave that initiates the neuroepithelial cell to neuroblast transition. EGF signalling drives the wave forward, while JAK/STAT signalling slows the progression of the wave [7, 9, 42] and prevents ectopic neuroblast formation in the epithelium [43]. PII progenitor cells secrete the EGF ligand, Spitz, which activates the EGFR pathway in neighbouring lateral PI progenitor cells. These neuroepithelial cells are positive for the EGFR downstream target gene *pointed P1* (*pntP1*). *pnt* or *spitz* loss-of-function mutant clones do not upregulate L’sc, indicating that both the induction of the proneural wave, and its progression, are downstream of EGFR signalling [7]. Moreover, the EGF signalling controls the levels of Dl ligand, regulating Notch signalling and the progression of the proneural wave [7, 44].

L’sc acts in a backward relay mechanism to induce the PI progenitor state. It induces the expression of Neur in PII progenitors and thus activates Delta-Notch signalling to induce PI. As a result neighbouring PI progenitors upregulate the Notch target gene E(spl)mγ. One role of high Notch signalling activity in PI is to induce cell cycle arrest in PI progenitor cells [19]. Hence, the backward relay mechanism controls the sequential and timely acquisition of progenitor states.

In order for neuroepithelial cells to transform into neuroblasts, Notch signalling must be blocked. Binding of Dl to Notch in the same cell can inhibit Notch signalling through a mechanism called ‘*cis*-inhibition’ [45]. We observed high Dl levels in PII progenitor cells where E(spl)mγ levels are low. Furthermore, we show that Dl misexpression clones show no Notch signalling activity. Therefore, it is plausible that Dl activates Notch in *trans*, inducing the PI progenitor state, while inhibiting Notch in *cis* to enable the progression from PII progenitors to neuroblasts.

### Notch signalling regulates stem cell heterogeneity from flies to vertebrates

The Notch signalling pathway regulates stem cell maintenance, proliferation and differentiation in different tissues, contributing to vertebrate development and organ regeneration. However, the effect of Notch signalling is highly dependent on the biological context [10]. During development and adult neurogenesis, NSCs are a highly heterogeneous population. NSCs can be found in proliferative or quiescent states. Furthermore, adult NSCs generate intermediate progenitor states with different potency before differentiation into neurons or glial cells [46]. Notch signalling preserves NSC maintenance and proliferation [47–49] and can also induce the quiescence state [14, 50–52]. The context of Notch signalling in NSCs determines the outcome. For example in zebrafish, whereas the Notch3 receptor induces a quiescence state in NSCs, Notch1b is required for NSC population maintenance [53]. This phenomenon resembles the different responses to Notch signalling in neuroepithelial cells in the *Drosophila* optic lobe.

Notch signalling interaction with other pathways also regulates NSC behaviour in the vertebrate brain. EGFR signalling in neural progenitors non-autonomously blocks Notch signalling in NSCs, reducing NSC proliferation at the adult subventricular zone [54]. Interestingly, EGFR is a downstream target of Notch signalling in NSCs [48], suggesting that Notch promotes both NSC maintenance and the formation of neural progenitors.

## Conclusions

Our study proposes a model of dynamic Notch signalling in the transition from neuroepithelial cells into neuroblasts. During *Drosophila* optic lobe development, Notch signalling regulates NSC amplification and maintenance in a similar manner to vertebrate NSCs. Notch signalling also induces the progression into PI/PII progenitor states. Understanding the dynamic regulation of Notch signalling during NSC state transitions in the optic lobe may yield new insights into the mechanisms that control adult neurogenesis and brain regeneration.

### Aknowledgements

We would like to thank Sarah Bray, François Schweisguth, Eugenia Piddini, Pat Simpson, Yuh Nung Jan, Jim Skeath and DSHB for antibodies and fly stocks. We thank Takumi Suzuki and Carlos Oliva for comments on the manusctript.

## Additional files

Additional file 1: **Figure S1.** *neur* is expressed in the transition zone. in situ hybridisation of larval brains using specific antisense probes against (A) *tom* and (B) *neur*. Note that *neur* and *tom* are expressed in a complementary pattern. (PSD 81600 kb)

Additional file 2: **Figure S2**. L’sc misexpression clones delaminate and induce ectopic transition zones in wild-type neuroepithelial cells. (A-A”) L’sc misexpressing clone that has delaminated from the neuroepithelium induces an ectopic transition zone. Staining for (A,A’) β-gal/*neur* in green, (A,A”) Dpn in red and (A) Notch receptor in gray. Clone is marked by GFP expression in blue. Note that the clone has delaminated inside the optic lobe and the ectopic transition zone has continued generating NBs. (PSD 12135 kb)

## Abbreviations

Ase: Asense
Dl: Delta
Dpn: Deadpan
L’sc: Lethal of scute
N: Notch
NB: Neuroblast
NE: Neuroepithelial
Neur: Neuralized
NICD: Notch intracellular domain
NSC: Neural stem cell
